# Unique Population or Unique Species? Genetic Insights Into the Pygmy Freshwater Crocodiles of Northern Australia

**DOI:** 10.1002/ece3.74055

**Published:** 2026-07-31

**Authors:** Katherine Brittain, Torre Muhlbach, Rui Cao, Jose Luis Mijangos, Dean Yibarbuk, Erin O'Brien, Ruchira Somaweera, Nancy N. FitzSimmons, Jaime Gongora

**Affiliations:** ^1^ Sydney School of Veterinary Sciences, Faculty of Science University of Sydney Sydney New South Wales Australia; ^2^ Institute for Applied Ecology University of Canberra Bruce Australian Capital Territory Australia; ^3^ Diversity Arrays Technology Pty Ltd University of Canberra Bruce Australian Capital Territory Australia; ^4^ Warddeken Land Management Ltd Nightcliff Northwest Territories Australia; ^5^ Wildlife Coexistence Howard Springs Northwest Territories Australia; ^6^ Ecology & Environmental Approvals (EEA) unit WSP Perth Western Australia Australia; ^7^ School of Environmental and Conservation Sciences Murdoch University Murdoch Western Australia Australia; ^8^ Australian Rivers Institute Griffith University Nathan Queensland Australia

**Keywords:** *Crocodylus johnstoni*, dd‐RADseq, dwarfism, phenotypic plasticity, speciation, wildlife conservation

## Abstract

Isolated populations of Australian freshwater crocodiles ( *Crocodylus johnstoni* ) in few elevated, rocky escarpment areas of the Northern Territory of Australia reach sexual maturity earlier and generally have an adult size half that of their downstream counterparts. There is debate over whether these “pygmy” or “dwarf” crocodile populations are the result of extreme phenotypic plasticity or the origins of a unique species through peripatric speciation. This debate is of conservation concern as these populations face immediate threats from invasive species and climate change, and species classification can affect conservation management strategies. We used mitochondrial DNA control region and cytochrome b sequences to investigate whether mtDNA haplotype variation supports any evidence of speciation between the pygmy and standard‐size freshwater crocodiles. Additionally, we used genotyping by sequencing (dd‐RADseq) to examine genetic clustering. Separated and concatenated control region and cytochrome b haplotypes were shared between pygmy and standard‐size crocodile populations. Principle Component Analysis and STRUCTURE analyses on dd‐RADseq data showed pygmy and standard‐size crocodiles cluster by geographic location as much as by phenotype. We found no clear and objective genetic evidence to suggest that the pygmy freshwater crocodiles should be considered a separate species from any other population of the standard‐size crocodiles. However, the isolation and environmental adaptability of these unique pygmy freshwater crocodile populations make them ecologically unique and valuable for conservation, and worth safeguarding against potential threats.

## Graphical abstract

There is debate over whether isolated populations of “pygmy” Australian freshwater crocodiles are a unique species. This is a conservation concern as these populations face unique challenges. We found some population differences which support these populations as being ecologically unique and valuable for targeted conservation.

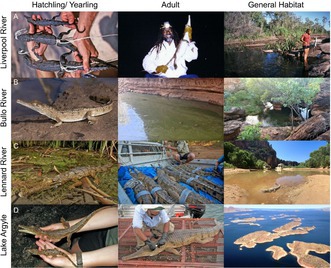

## 1 Introduction

The genus *Crocodylus* (i.e., true crocodiles) makes up approximately half of the extant diversity of the Order Crocodylia (crocodylians) (Oaks 2011; Grigg 2015; Uetz et al. 2025). True crocodiles span a diverse range of habitats, with three major evolutionary lineages distributed in Australasia, the Americas, and Africa (Meredith et al. 2011; Oaks 2011). Two species of crocodylians occur in Australia, which are sympatric in parts of northern Australia. The larger of the two, the saltwater or estuarine crocodile ( *Crocodylus porosus* ), inhabits possibly all tidal waterways and many adjacent freshwater systems across tropical northern Australia, as well as small islands offshore (Webb et al. 1983). The smaller Australian freshwater crocodile ( *C. johnstoni* ) is endemic to tropical mainland northern Australia (in Western Australia, WA; Northern Territory, NT; and Queensland, QLD) and has not been reported from any offshore islands. Introduced populations exist from Townsville to Rockhampton in QLD and further south (Read et al. 2004). They typically inhabit freshwater habitats upstream of tidal influence within their range, while few populations extend into tidal, saline waters (Webb et al. 1983; Read et al. 2004). The males of this species can grow to over 3 m in length and the females over 2 m, and maximum recorded ages in the wild are 64 years for males and 54 years for females (Webb and Manolis 1989; Tucker et al. 2006).

**FIGURE 1:**
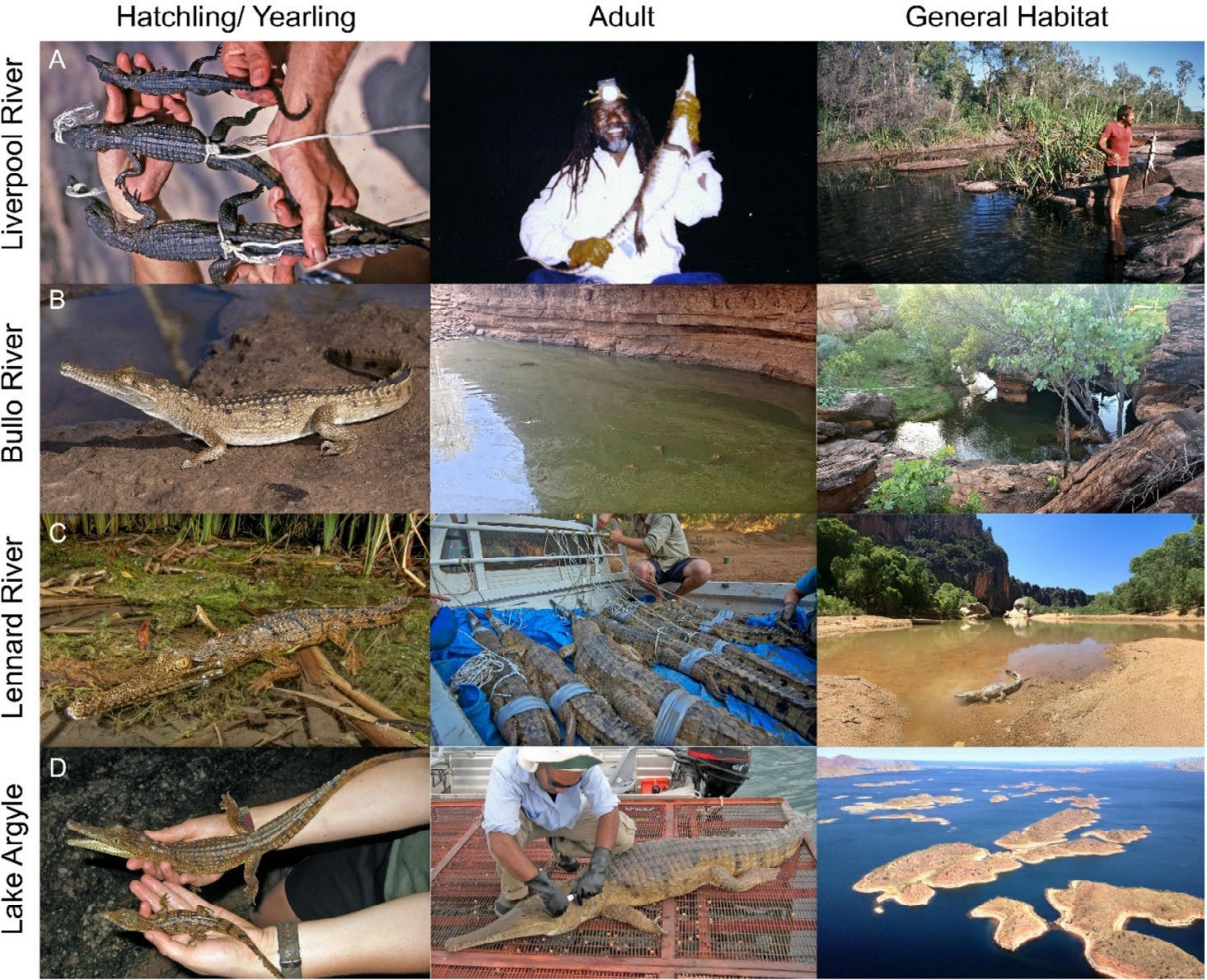
Variation in morphology and habitat characteristics of crocodiles across different regions, illustrating differences in coloration, relative size, and condition. (A) Upper escarpments of the Liverpool River, Northern Territory, where individuals were often emaciated and appeared “old”, including a gravid female held by Dean Yibarbuk (photos: Grahame Webb; Nancy Fitzsimmons); (B) Upper escarpment of the Bullo River, Northern Territory, where crocodiles are relatively small but not notably emaciated (photos: Angus McNab and Eridani Mulder); (C) Seasonally flowing Leonard River, Western Australia, where large numbers of crocodiles congregate in isolated pools during the dry season, and adults reach approximately 2 m in total length (photos: Ruchira Somaweera); and (D) Permanent and deep waters at Lake Argyle, Western Australia, where consistent food and resource availability allow adults to attain lengths approaching or exceeding 3 m (photos: Ruchira Somaweera).

In the 1980s, isolated populations of freshwater crocodiles were found in higher‐elevation, escarpment habitats at the Liverpool River in Arnhem Land (NT) that exhibit several morphological trait variations compared to the standard‐size freshwater crocodile (Figure 1; Webb 1985). These crocodiles generally grow to half the mature adult size of their downstream counterparts and reach sexual maturity at a smaller size (Webb 1985). Consequently, these populations are referred to as “dwarf,” “stunted,” “stone country,” or “pygmy” (hereafter pygmy) crocodiles by the popular media (Britton et al. 2013). A second population was reported in the upper reaches of the Bullo River (NT) in the 2000s that may have greater size variation, with the largest crocodile found at a total length of 1.7 m (Figure 1; Britton et al. 2013).

There has been a public debate as to whether these pygmy freshwater crocodile populations are an example of phenotypic plasticity—the varying phenotypic expression of an organism in response to environmental stimuli or resource availability (West‐Eberhard 2005; Pfennig and West‐Eberhard 2021) or whether speciation has occurred (Webb 1985; McCue 2014; Webber 2015). More than 30 species concepts have been proposed, each emphasizing different criteria—including morphological, biological, ecological, or genetic characteristics—for delineating species boundaries (reviewed in Zachos 2016). Each of these frameworks has its own strengths and limitations, with their applicability often depending on the taxonomic group and research context. More recent approaches increasingly adopt integrative frameworks that synthesize multiple species concepts, typically unified by the recognition of species as separately evolving evolutionary lineages (Mayden 1999; De Queiroz and Weins 2007; Naomi 2011). A very limited amount of peer‐reviewed research in ecology, biology or genetics exists in quantifying differences in pygmy freshwater crocodiles. Morphometric analyses have revealed overall reduced dimensions in pygmy populations compared to other freshwater crocodiles (Edwards et al. 2017). However, geographic variation in morphology is common among crocodylians (Webb and Messel 1978; Nestler 2012; Labarre et al. 2017). Because the pygmy and standard‐size freshwater populations are allopatric, the biological species concept, which relies on testing reproductive compatibility, cannot be applied in this context. In contrast, the genetic species concept, which defines species as groups of interbreeding populations that are genetically isolated from others (Baker and Bradley 2006), may offer more suitable insights into crocodylian speciation.

Applying the genetic species concept relies on mitochondrial molecular markers such as the control region (CR) and cytochrome b (Cytb), and nuclear markers such as single nucleotide polymorphisms (SNPs) to provide information on neutral and adaptive evolution, and diversification among and within populations. For this study, we have chosen to investigate CR due to its high mutation rate, making it useful for determining intraspecific relationships and its common use in the literature to report population genetics for other crocodylian species. The Cytb gene was chosen to provide resolution at an inter‐specific level, as it is more commonly used to report genetic relationships between species. SNP data generated through the whole genome reduced representation sequencing approach (dd‐RADseq) was also used on a smaller subset of individuals to generate a high‐resolution dataset, as crocodylian mitochondrial genetic diversity can be low (Luck et al. 2012; Afsharian et al. 2018).

While classifying what constitutes a species is an ongoing discussion (Baker and Bradley 2006; Hillis 2019) and beyond the scope of this paper, our study aims to produce genetic evidence to assist in making conservation and management decisions. Classifying the pygmy freshwater crocodiles as a distinct species from the standard‐size freshwater crocodile could have important implications for the conservation management of these populations. Currently, the freshwater crocodile is listed as “Least Concern” on the IUCN Red List of Threatened Species, based on the total population size of the species, largely intact habitat and a wide distribution (Isberg et al. 2017). However, this would likely change for the pygmy populations if they were considered genetically distinct. Our objectives for this study were to (1) investigate the genetic variation and lineage diversification of the pygmy and standard‐size freshwater crocodiles from representative and widely spread sites across northern Australia using the hypervariable mitochondrial CR and Cytb sequences; and (2) investigate the genetic clustering and relatedness statistics of pygmy and standard‐size freshwater crocodiles across a subset of the species distribution to assess current levels of gene flow and speciation potential of freshwater crocodile populations. We hypothesize that genetic markers will differentiate pygmy from standard‐sized freshwater crocodiles, providing support for targeted conservation and management practices for pygmy populations.

## 2 Materials and Methods

### 2.1 Sample Collection and DNA Extraction

Freshwater crocodile tissues were collected from 21 sampling locations across the species' distribution in the river systems of northern Australia, including samples from pygmy freshwater crocodile populations (Table A1). All samples were collected for other studies and appropriated for this paper (FitzSimmons et al. 2000; Somaweera and Shine 2011; Somaweera et al. 2013, 2019; Cao et al. 2020). In summary, individuals were captured and restrained using methods appropriate to their size (Cao et al. 2020), and dorsal tail scutes were cut using scissors or a knife dipped in 70% ethanol for marking the crocodiles, and the resulting tissues stored in > 90% ethanol. All methods were approved by the CSIRO Animal Ethics Committee (AEC) (approvals 2017–18 and 2017–22) and the Department of Biodiversity Conservation and Attractions AEC (approval 2014/13), with relevant scientific research permits 08‐000949‐1, 08‐000950‐1, SC001387 and ECNR970004. DNA was extracted from the samples using the DNeasy Blood and Tissue extraction kit from Qiagen (Germany), the phenol‐chloroform protocol (Green and Sambrook 2012) or the Isolate II Genomic DNA kit from Bioline. The concentration and quality of DNA samples were determined using Qubit (Life Technologies, USA), Nanodrop and 1% agarose gels.

### 2.2 Analysis of Control Region and Cytochrome b

To examine genetic distances and haplotype sharing between pygmy and standard‐size freshwater crocodile populations, we used 85 of the collected samples to analyse two mtDNA markers. A 794 bp fragment of the mtDNA CR was amplified using primers L15463 and 5′ CAC TAA AAT TAC AGA AGA GCC GAC 3′ modified for freshwater crocodile specificity from universal crocodylian primer H16260 in FitzSimmons et al. (2002) and the following PCR thermal cycling conditions: initial denaturation for 2 min at 94°C, 32 cycles of denaturation at 94°C for 25 s, annealing at 48°C for 45 s and extension at 72°C for 45 s, followed by one cycle of 72°C for 5 min for the final extension. A 1347 bp fragment of the mtDNA Cytb gene including flanking regions was amplified using primers 5′ ACC AAG ACC TAG GGC ACG AAA AAC C 3′ and 5′ TCT GTC TTA CAA GGC CAG CGC TTT 3′ that were modified for freshwater crocodile specificity from universal crocodylian primers CP14126 and CP15546 in Meganathan et al. (2009). PCR conditions were: 94°C for 5 min of initial denaturation followed by 30 cycles of denaturation at 95°C for 1 min; annealing at 50°C for 30 s; extension at 72°C for 30 s. Amplification ended with a 5 min final extension step. Samples were sent to the Australian Genome Research Facility (AGRF) for PCR amplification and Sanger sequencing.

Sequence reads were aligned using ClustalW (Thompson et al. 1994) and trimmed in BioEdit (Hall 1999) to give a final sequence length of 685 bp for the CR and 1200 bp for the Cytb gene. Additionally, 18 CR sequences from 18 Bullo River pygmy freshwater crocodiles were aligned with CR data generated here. As some sites only had one or two samples, sites were grouped into river basins for further analysis, except for the Bullo River samples (Victoria River Basin), which were analyzed independently of the Victoria River Basin standard‐size samples. We assessed whether the level of divergence among haplotypes found in standard‐size versus pygmy freshwater crocodile phenotypes was greater than the level of divergence among haplotypes found in different standard‐size crocodile river basin populations. This was done by calculating pairwise genetic distances of haplotypes between and within river basin populations for each of the CR and Cytb haplotype sets in MEGA‐X (Kumar et al. 2018) using the Tamura‐Nei substitution model. This model was chosen as the best fit for CR and Cytb analyses based on the Bayesian information criterion given in MEGA‐X. To visualise the shared and unique haplotype distribution among river basins, median joining networks (MJN) and haplotype maps were created in PopART 1.7 (Leigh and Bryant 2015). The CR and Cytb sequences (excluding Bullo River samples; *n* = 85) were then concatenated to give a single alignment, and an additional MJN was created for these data.

### 2.3 Population Clustering Using Genotyping by Sequencing dd‐RADseq

To generate dd‐RADseq data, seven of the pygmy freshwater crocodile samples from Liverpool River used in the mtDNA analysis and one additional pygmy freshwater crocodile sample from Liverpool River were used. Methods and analyses followed that of Cao et al. (2020), who analysed standard‐size freshwater crocodiles in the east and west Kimberley region of Australia. Genomic DNA (200 ng) was double‐digested using the enzyme pair ecoRI and NlaIII, with the ligation‐compatible barcode adapters A and P2, and restriction site overhang. Fragmented DNA was enriched by PCR, size selected for 60 bp, and sequenced using the NextSeq platform (Illumina, USA) in four lanes. SNP discovery was done using a maximum likelihood model implemented in Stacks (Catchen et al. 2013) before being stored in a VCF file as described in Cao et al. (2020). To improve the quality of our analyses, the standard‐size crocodile data (stored in the Sequence Read Archive under project number PRJNA551392) used in Cao et al. (2020) was refiltered along with the pygmy freshwater crocodile data as a single dataset. The dataset was filtered in R using packages from the dartRverse (Gruber et al. 2018; Mijangos et al. 2022) to exclude individuals with more than 75% missing data and loci missing from more than 70% of individuals. These thresholds were chosen due to the overall poor SNP call rate of the dataset and to keep the small number of pygmy freshwater crocodile samples in the dataset. Loci with a minor allele frequency below 0.05 were removed, as were loci in linkage disequilibrium above the threshold of *r* ^2^ = 0.2. The dataset was also filtered in dartRverse to remove SNPs out of Hardy–Weinberg equilibrium in all sampling locations using the “Out‐All” approach (Pearman et al. 2022) using a *p*‐value of 1 × 10^−10^ adjusted for multiple comparisons and other default parameters.

To measure genetic variation within sample locations (), observed heterozygosity (*H*_*o*_), unbiased (i.e., corrected for sample size) expected heterozygosity (*uH*_e_) and inbreeding coefficient (*F*_*IS*_) were calculated. Expected heterozygosity was tested for significant difference (*p* < 0.05) between sampling locations. To visualize spatial patterns of genetic variation in freshwater crocodiles, principal component analysis (PCA) was performed using the dartRverse.

Fixed differences (i.e., where two populations share no alleles) were used as indicators of gene flow (Georges et al. 2018) among freshwater crocodile sampling locations and calculated using the dartRverse. Fixed differences were used to infer operational taxonomic units (OTUs) by iteratively collapsing populations with more than one fixed difference following Georges et al. (2018). To account for sample‐size bias, we estimated the expected number of false‐positive fixed differences by simulation conditional on group sizes and allele‐frequency profiles and tested observed counts for significance (test = TRUE; *α* = 0.01).

Inbreeding (mating of related individuals) is one factor that can increase genetic differentiation between populations, particularly if populations are small (Huang et al. 2009). To investigate the degree to which inbreeding may have influenced genetic structure in freshwater crocodile populations, the probability of identity by descent (IBD) was calculated across all loci that would result from all the possible crosses of the individuals that were sampled. IBD was estimated by an additive relationship matrix approach (Endelman and Jannink 2012) as implemented in dartRverse and rrBLUP (Endelman 2011). To further investigate levels of inbreeding, inbreeding coefficients were estimated for each individual using two different statistics as described in Keller et al. (2011). These were (a) F_alt_, where a homozygous locus in an individual is weighted by the inverse of that allele's frequency in the population using the software GCTA (Yang et al. 2011) and the command—ibc, and (b) F_h_, which is a deviation in homozygosity from its Hardy–Weinberg expectation using the software PLINK (Purcell et al. 2007) using the—het command (Earl and vonHoldt 2012).

Population clustering was further investigated using STRUCTURE (Pritchard et al. 2000). The “no admixture” model with default settings was run with 50,000 burn‐in steps and 50,000 replications for five iterations for each K (number of populations modelled) from 1 to 6. The most likely K was selected using the delta K method described by Evanno et al. (2005) and supported by the Ln Pr(X|K) method described by Pritchard et al. (2000). Plots for these methods were generated in Structure Harvester (Earl and vonHoldt 2012). To determine substructure, the STRUCTURE model was repeated on the subset of data for each K found until no clear structure was identified (Pritchard and Wen 2002). Final structure plots were generated using CLUMPP (Jakobsson and Rosenberg 2007) and DISTRUCT (Rosenberg 2004).

Genetic differentiation among sampling locations was estimated using the fixation index (*F*_*ST*_), calculated according to Nei (1987; equation 7.36) as implemented in the dartRverse. Additionally, we determined the partitioning of genetic variation across hierarchical levels using analysis of molecular variance (AMOVA; Excoffier et al. 1992) using the function poppr.amova from the R package poppr (Kamvar et al. 2014). To estimate variance components and associated Φ‐statistics, we used the ade4 implementation (Dray and Dufour 2007). Statistical significance was evaluated by permutation tests with 9999 randomizations.

## 3 Results

### 3.1 Mitochondrial Cytochrome b and Control Region Analyses

**FIGURE 2:**
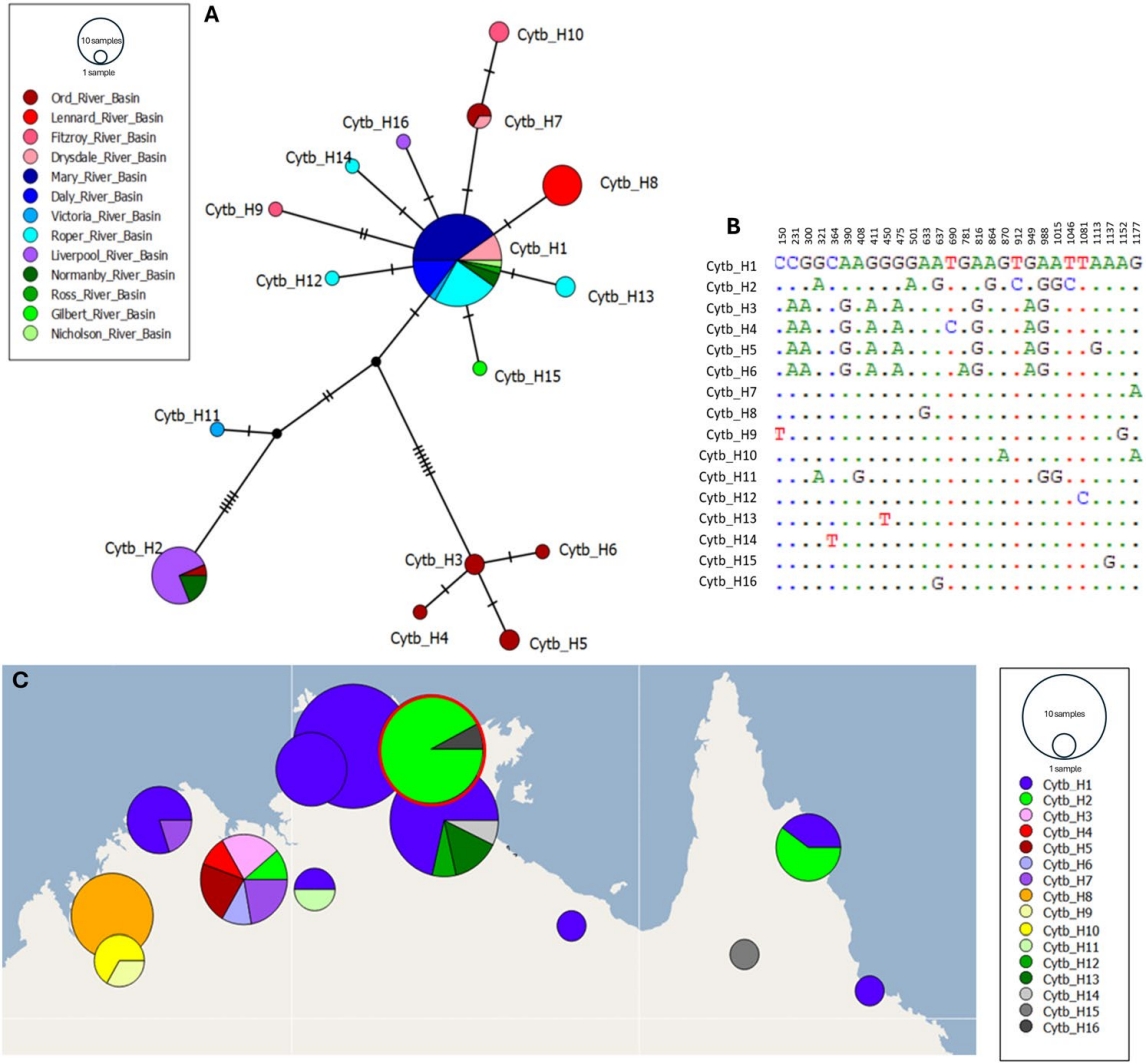
Haplotypes of Cytochrome b in 85 freshwater crocodiles across 13 river basins in Northern Australia. (A) Median joining network of the 16 haplotypes, mutations between haplotypes are represented by a dash; (B) SNP positions within the cytochrome b haplotypes; and (C) distribution map of the haplotypes. The pygmy population at Liverpool River Basin is outlined in red. This figure was generated using PopART1.7.

**FIGURE 3:**
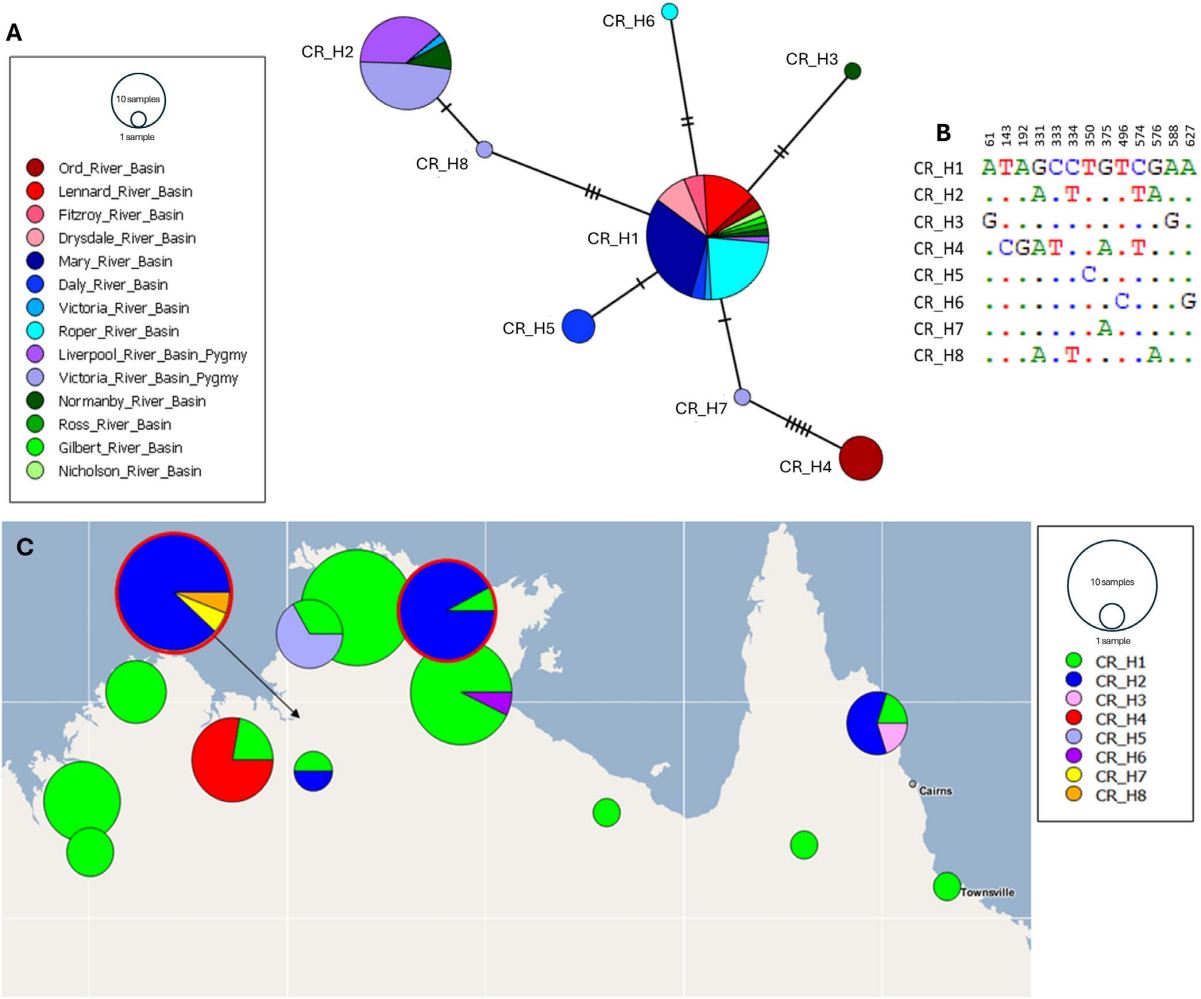
Haplotypes of Control Region in 103 freshwater crocodiles across 13 river basins in Northern Australia (Victoria River Basin split by pygmy and standard phenotype). (A) Median joining network of the eight haplotypes, mutations between haplotypes are represented by a dash; (B) SNP positions within the Control Region haplotypes; and (C) distribution map of the haplotypes. The pygmy populations at Liverpool and Victoria River Basins are outlined in red. This figure was generated using PopART1.7.

Shared and unique Cytochrome *b* (Cytb) and Control Region (CR) haplotypes were found between the pygmy and standard‐size freshwater crocodile populations (stored in BankIt with GenBank accession numbers PV102152 to PV102236 and PV102049 to PV102151 respectively). The partial Cytb gene contained 28 substitutions to give 16 distinct haplotypes, and the partial CR sequence contained 13 substitutions to give eight distinct haplotypes. The Cytb haplotypes were labelled Cytb_H1 through Cytb_H16 (Figure 2). Haplotype Cytb_H1 was not found in pygmy freshwater crocodiles but was found in standard‐size crocodiles in eight of 13 river basins in Western Australia (WA), Queensland (QLD), and the Northern Territory (NT) (Figure 3). Cytb_H2 was differentiated from Cytb_H1 by eight mutations and was the predominant haplotype in the Liverpool River Basin pygmy freshwater crocodile population. This haplotype was also found in standard‐size individuals in the Normanby River Basin (QLD) and Ord River Basin (WA). Haplotype Cytb_16 was identified only in the pygmy freshwater crocodile samples and differed from Cytb_H1 by a single mutation. Of the remaining 13 haplotypes, 12 were unique to their river basin, and only one was found across two West Australian river basins. The MJN analysis did not suggest any clear clustering patterns among haplotypes and geographical location, except for a cluster of haplotypes (Cytb_H3‐6) found only in the Ord River Basin that differed by at least seven mutations from any other haplotype.

The CR haplotypes were labelled CR_H1 through CR_H8. The CR haplotypes, including those from the Bullo River samples, showed the same pattern as the Cytb haplotypes of having one main haplotype. Haplotype CR_H1 was found in 12 of 14 river basins across the entire geographical range, including the Liverpool River Basin pygmy freshwater crocodile samples. CR_H2 was found in 4 of 12 river basins across the Northern Territory and Queensland, including the Bullo River and Liverpool River Basins. The remaining six haplotypes were unique to a single river basin. Of the 18 Bullo River samples, 16 had the CR_H2 haplotype, and two had unique haplotypes (CR_H7, CR_H8). The Liverpool River Basin pygmy freshwater crocodiles had no unique haplotypes. The MJN did not show any clear clustering patterns among haplotypes and geographical locations.

Pairwise genetic distances between CR haplotypes in pygmy and standard‐size freshwater crocodiles ranged from 0.15% to 1.21% (Table 1). The greatest pairwise distance occurred between haplotypes unique to standard‐size freshwater populations in the Ord River Basin (WA) versus Normanby River Basin (QLD) or Roper River Basin (NT). Pairwise distances between the Cytb haplotypes ranged from 0.08% to 1.28%. The greatest pairwise distances (> 1.19%) occurred between Cytb_H2, found in pygmy and standard‐size freshwater crocodiles, and haplotypes Cytb_H3–H6, which were all haplotypes unique to the Ord River Basin (WA).

**TABLE 1:** Pairwise distances of mtDNA markers in populations of Australian freshwater crocodiles.

**Cytb_H2**	0.0067							**CR_H2**	**CR_H3**	**CR_H4**	**CR_H5**	**CR_H6**	**CR_H7**	**CR_H8**	
**Cytb_H3**	0.0068	0.0119						0.0060	0.0030	0.0090	0.0015	0.0030	0.0015	0.0045	**CR_H1**
**Cytb_H4**	0.0076	0.0127	0.0008						0.0091	0.0090	0.0075	0.0090	0.0075	0.0015	**CR_H2**
**Cytb_H5**	0.0076	0.0128	0.0008	0.0017						0.0121	0.0045	0.006	0.0045	0.0076	**CR_H3**
**Cytb_H6**	0.0076	0.0128	0.0008	0.0017	0.0017						0.0106	0.0121	0.0075	0.0106	**CR_H4**
**Cytb_H7**	0.0008	0.0076	0.0076	0.0085	0.0085	0.0085						0.0045	0.0030	0.0060	**CR_H5**
**Cytb_H8**	0.0008	0.0076	0.0076	0.0085	0.0085	0.0085	0.0017						0.0045	0.0075	**CR_H6**
**Cytb_H9**	0.0017	0.0084	0.0085	0.0093	0.0093	0.0093	0.0025	0.0025						0.006	**CR_H7**
**Cytb_H10**	0.0017	0.0085	0.0085	0.0093	0.0094	0.0094	0.0008	0.0025	0.0034						
**Cytb_H11**	0.0034	0.0050	0.0085	0.0093	0.0094	0.0094	0.0042	0.0042	0.005	0.0051					
**Cytb_H12**	0.0008	0.0076	0.0076	0.0084	0.0085	0.0085	0.0017	0.0017	0.0025	0.0025	0.0042				
**Cytb_H13**	0.0008	0.0076	0.0076	0.0084	0.0085	0.0085	0.0017	0.0017	0.0025	0.0025	0.0042	0.0017			
**Cytb_H14**	0.0008	0.0076	0.0076	0.0084	0.0085	0.0085	0.0017	0.0017	0.0025	0.0025	0.0042	0.0017	0.0017		
**Cytb_H15**	0.0008	0.0076	0.0076	0.0085	0.0085	0.0085	0.0017	0.0017	0.0025	0.0025	0.0042	0.0017	0.0017	0.0017	
**Cytb_H16**	0.0008	0.0059	0.0076	0.0085	0.0085	0.0085	0.0017	0.0017	0.0025	0.0025	0.0042	0.0017	0.0017	0.0017	0.0017
	**Cytb_H1**	**Cytb_H2**	**Cytb_H3**	**Cytb_H4**	**Cytb_H5**	**Cytb_H6**	**Cytb_H7**	**Cytb_H8**	**Cytb_H9**	**Cytb_H10**	**Cytb_H11**	**Cytb_H12**	**Cytb_H13**	**Cytb_H14**	**Cytb_H15**

*Note:* Pairwise distances are shown for Cytochrome b (below the diagonal) and for partial control region (above the diagonal) for populations of pygmy and standard sized freshwater crocodiles across northern Australia. Distances were generated using the Tamura‐Nei model of nucleotide substitution in MEGA‐X. Shaded cells represent row/column headings, as numbers in the table represent two sets of data above and below the diagonal.

### 3.2 Population Clustering Using dd‐RADseq

**FIGURE 4:**
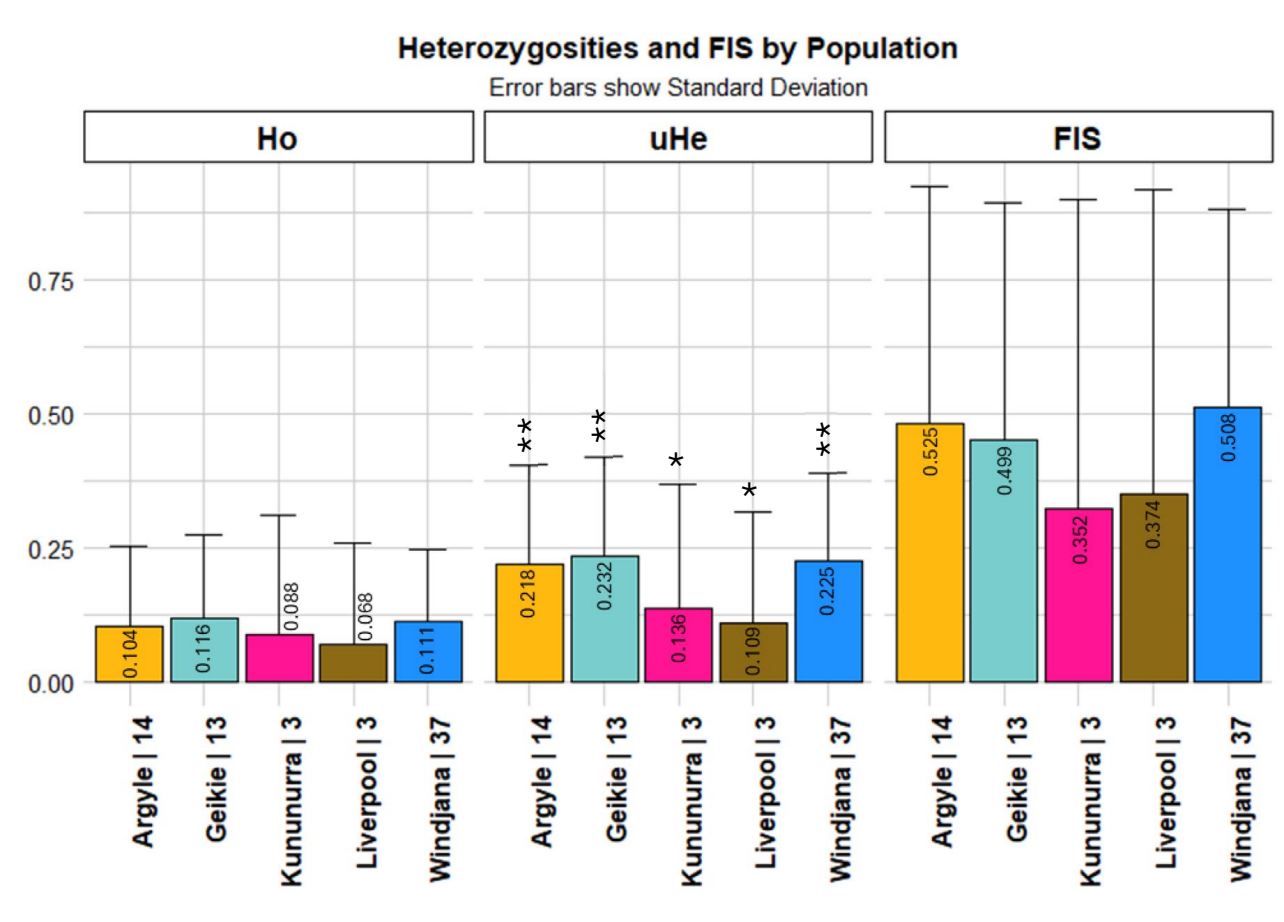
Measures of genetic diversity in standard and pygmy freshwater crocodiles. Genetic diversity was measured with observed heterozygosity (Ho), unbiased expected heterozygosity (uHe), and inbreeding coefficient (FIS). uHe marked with (*) were significantly different from sites marked with (**) but not from each other. Samples from Windjana Gorge (Lennard River Basin) and Geikie Gorge (Fitzroy River Basin) are from the west Kimberley; Lake Argyle and Lake Kununurra (Ord River basin) are from the east Kimberley, and pygmy crocodile samples are from the Liverpool River Basin in the Northern Territory. Labels at the bottom of the figure contain the group name and the mean sample size per locus per group.

Sequencing and variant calling generated 88,672 loci across 181 individuals. After filtering, we retained 172 individuals and 1032 loci for downstream analyses. Genetic diversity as measured by observed heterozygosity (*H*_*o*_) was similar among sample locations, while unbiased expected heterozygosity (*uH*_*e*_) varied between locations (Figure 4). *H*_*e*_ was lower in the groups sampled at Liverpool River (pygmy freshwater crocodiles) and at Lake Kununurra, which also had a small sample size per locus. *H*_*e*_ was not significantly different between Liverpool River and Lake Kununurra samples, but was significantly different between each of those two sites and all other locations. Inbreeding coefficient *F*_*IS*_ was positive in all sample locations.

**FIGURE 5:**
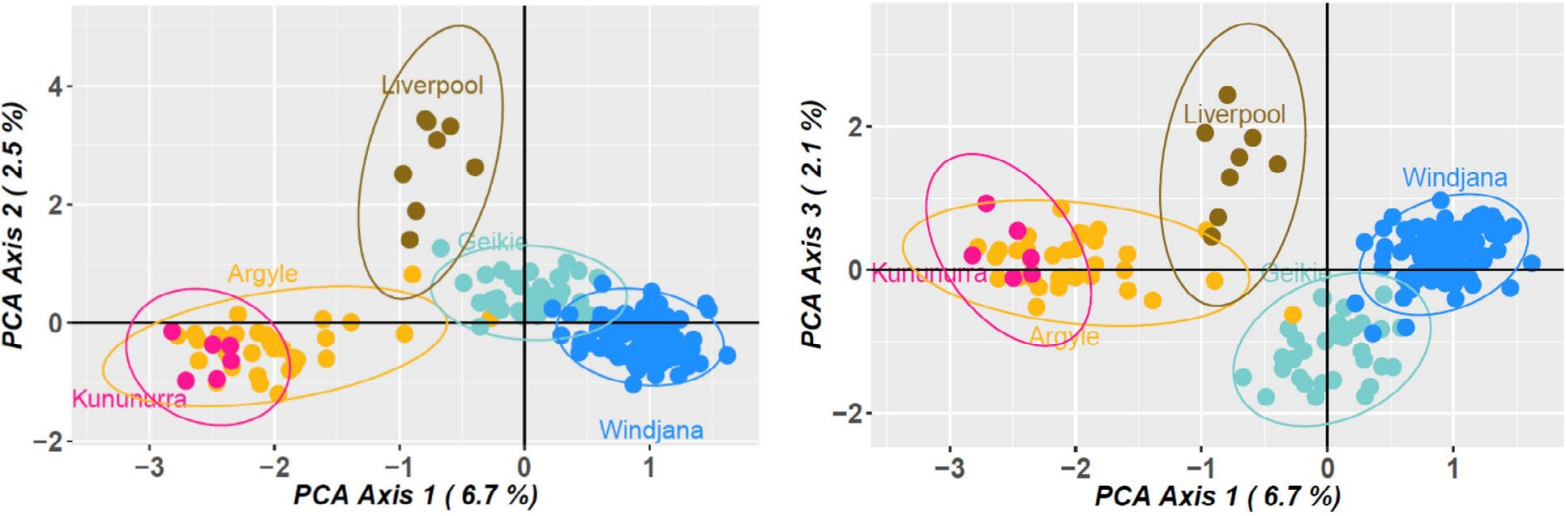
Principal component analysis (PCA) of dd‐RADseq data from Australian freshwater crocodiles. (A) First and second principal component axes; and (B) first and third principal component axes showing four different clusters. Samples from Windjana Gorge (Lennard River Basin) and Geikie Gorge (Fitzroy River Basin) are from the west Kimberley; Lake Argyle and Lake Kununurra (Ord River basin) are from the east Kimberley, and pygmy crocodile samples are from the Liverpool River Basin in the Northern Territory. Each dot represents one individual.

Principal component analyses (PCA) showed that the dd‐RADseq data segregated into four different clusters based on river basins (Figure 5). The first principal component axis (PC1) explained 6.7% of the variance between samples and separated Lakes Argyle and Kununurra (Ord River Basin) and Liverpool River (Liverpool River Basin) from Geikie Gorge (Fitzroy River Basin) and Windjana Gorge (Lennard River Basin), as well as showing a slight segregation between Geikie Gorge and Windjana Gorge. PC2 explained 2.5% of the variance and separated the pygmy individuals from the rest of the individuals in other groups, and PC3 explained 2.1% of the variance and separated individuals from Geikie Gorge from individuals sampled at Windjana Gorge. Combined, these three PC axes show segregation of samples by river basin, with some overlap.

The largest number of fixed differences, when each group was compared to the rest of the individuals (Table 2), was observed in the pygmy freshwater crocodiles, despite the small sample size. Fixed difference analysis grouped populations into three operational taxonomic units (OTU), which aligned with defined geographic areas of west Kimberley, east Kimberley, and Arnhem Land escarpments in the Northern Territory (Table 3). The observed number of fixed differences between these OTUs significantly exceeded expected false positives in all pairwise comparisons (*p* = < 0.001).

**TABLE 2:** Fixed differences by sampling location in one‐versus‐rest comparison.

Pop 1	Pop 2	Sample size Pop 1	Sample size Pop 2	Number of fixed differences
Argyle	Rest	33	140	0
Geikie	Rest	33	140	0
Kununurra	Rest	6	167	0
Liverpool	Rest	8	165	5
Windjana	Rest	93	80	0

*Note:* For each location, values are the number of loci fixed for alternate alleles in the focal location relative to all other individuals pooled.

**TABLE 3:** Fixed difference analysis of standard and pygmy sized freshwater crocodiles.

Operational taxonomic units (OTU)	Number fixed differences	Expected false positives	Standard deviation false positives	*p*
Western Kimberley vs. eastern Kimberley	14	0.5	0.36	< 0.001
Eastern Kimberly vs. Liverpool River Basin	27	9.6	1.79	< 0.001
Western Kimberley vs. Liverpool River Basin	26	4.8	1.3	< 0.001

*Note:* Analyses defined three “operational taxonomic units” (OTU) where different alleles of SNPs become fixed in populations of freshwater crocodiles.

**FIGURE 6:**
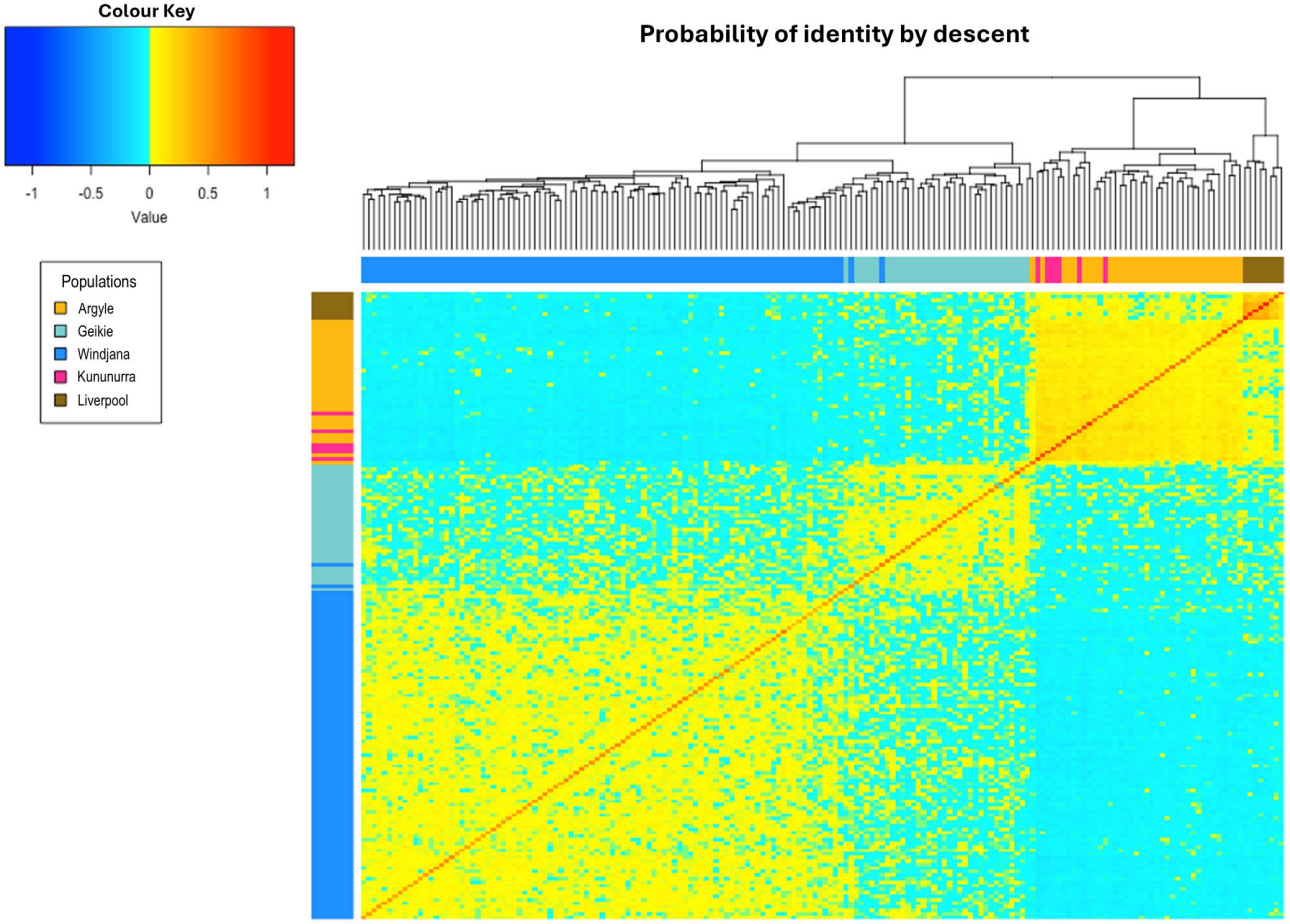
Heatmap of the probabilities of identity by descent (IBD) in freshwater crocodiles. Samples from Windjana Gorge (Lennard River Basin) and Geikie Gorge (Fitzroy River Basin) are from the west Kimberley; Lake Argyle and Lake Kununurra (Ord River basin) are from the east Kimberley, and pygmy crocodile samples are from the Liverpool River Basin in the Northern Territory. Yellow and red colours indicate those individuals that are more related to each other. The identification number of each individual is shown in the margins of the figure.

**FIGURE 7:**
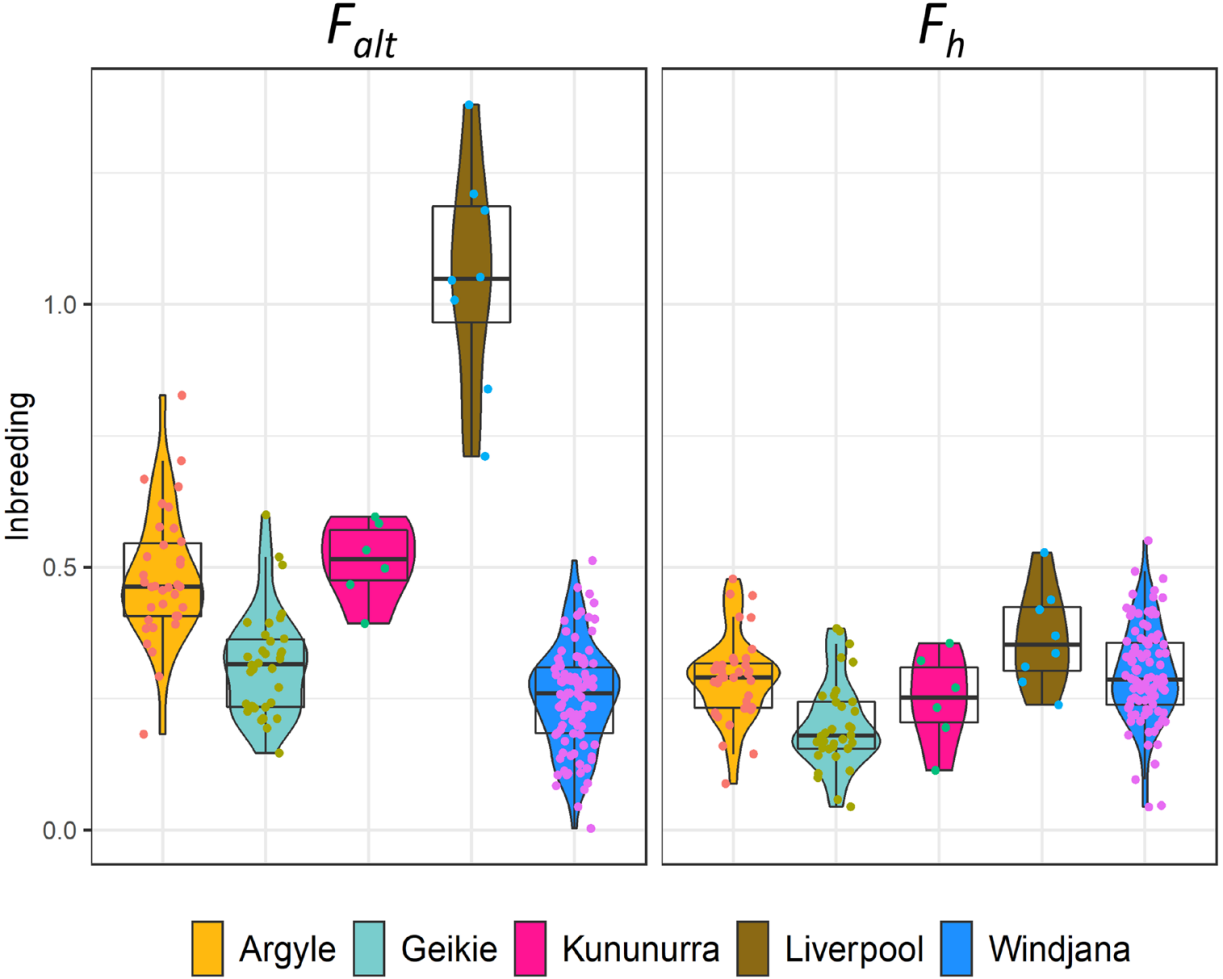
Violin plots showing estimates of inbreeding by individual in freshwater crocodiles. Falt, in which homozygous loci are weighted with the inverse of their allele frequency, was calculated using the software GCTA. Fh, the deviation in homozygosity from its Hardy–Weinberg expectation, was calculated using the software PLINK. Samples from Windjana Gorge (Lennard River Basin) and Geikie Gorge (Fitzroy River Basin) are from the west Kimberley; Lake Argyle and Lake Kununurra (Ord River basin) are from the east Kimberley, and pygmy crocodile samples are from the Liverpool River Basin in the Northern Territory. Each point represents one individual.

**FIGURE 8:**
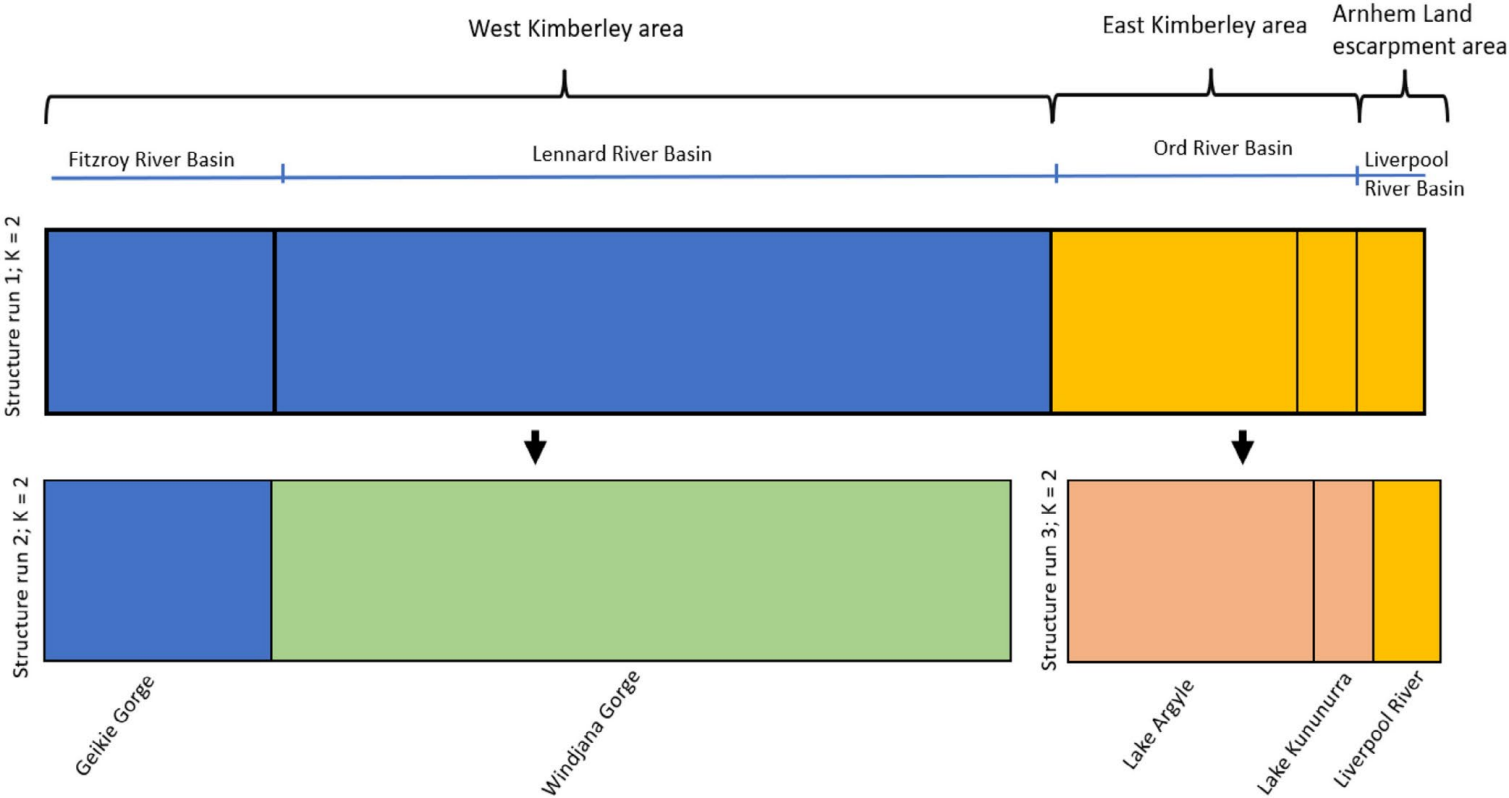
Sequential STRUCTURE graphs show genetic clustering of freshwater crocodile samples by river basin. Structure analyses did not detect genetic structure within river basins. Samples from the east and west Kimberley areas were from standard‐sized crocodiles, and samples from the Arnhem Land escarpment areas were taken from pygmy‐sized animals.

The dendrogram based on identity by descent (IBD) results (Figure 6) agreed with the results of the PCA analysis in identifying four clusters, each including related individuals. Pygmy freshwater crocodiles sampled in the Liverpool River had the highest IBD values. In agreement with IBD results, mean inbreeding coefficients by individual (Figure 7) were higher in the pygmy freshwater crocodile group sampled in the Liverpool River than in other groups, particularly in the statistic *F*_*alt*_. Clustering shown by sequential STRUCTURE analyses (Figure 8) using the DeltaK and Ln Pr(X|K) methods identified two genetic lineages (*K* = 2), separating samples by geography into the west Kimberley area, and the east Kimberley area plus Arnhem Lan escarpment area. Subsequent STRUCTURE analysis revealed substructure within each lineage, resulting in further clustering, which was consistent with the four river basins.

AMOVA results (Table 4) showed that most genetic variation was within individuals (67.38%; *σ* ^2^ = 41.19), followed by among individuals within populations (22.51%; *σ* ^2^ = 13.76), with a smaller component among populations (10.11%; *σ* ^2^ = 6.18). Φ‐statistics (Table 5) indicated moderate structure (Φ_ST = 0.3262, Φ_IS = 0.2504, Φ_IT = 0.1011), consistent with limited among‐population differentiation and appreciable inter‐individual variance within populations.

**TABLE 4:** Analysis of molecular variance (AMOVA; Excoffier et al. 1992) as implemented in the R package poppr (Kamvar et al. 2014) using 9999 permutations.

Source of variation	Degrees of freedom	Sum of squares	Mean squares	Sigma	Percentage
Among populations	4	1676.21	419.05	6.18	10.11
Among individuals within populations	173	11,885.59	68.70	13.76	22.51
Within individuals	178	7331.68	41.19	41.19	67.38
Total	355	20,893.48	58.85	61.13	100

*Note:* Sigma is the variance; Percentage is the percent of the total variance explained by each source of variance.

**TABLE 5:** Φ‐statistics.

Statistic	Definition	Estimate
Φ_ST	Among populations vs. total	0.3262
Φ_IS	Among individuals within populations	0.2504
Φ_IT	Individuals vs. total	0.1011

Genetic differentiation between sampling locations ranged from very low (Argyle–Kununurra: *F*_*ST*_ = 0.011) to high (Windjana–Liverpool: *F*_*ST*_ = 0.239), with generally high values for all pairwise comparisons involving Liverpool (0.203–0.239). In contrast, Argyle–Kununurra (0.011) and Windjana–Geikie were low (0.059), suggesting relatively high connectivity between these locations (Table 6).

**TABLE 6:** Pairwise FST among sampling locations following Nei (1987).

	Argyle	Geikie	Kununurra	Liverpool
Geikie	0.1215			
Kununurra	0.0111	0.1267		
Liverpool	0.2185	0.2029	0.2126	
Windjana	0.1766	0.0591	0.1841	0.2390

## 4 Discussion

We analyzed mitochondrial sequences and SNPs to inform the debate on whether the traits displayed by pygmy freshwater crocodiles in comparison to standard‐size freshwater crocodiles are due to phenotypic plasticity or a result of an ongoing speciation process. While genetic differentiation is occurring between Australian freshwater crocodile populations, we did not find strong evidence suggesting that speciation has occurred despite the phenotypic differences observed in the population of pygmy freshwater crocodiles in escarpment areas of the Liverpool River in Arnhem Land (Webb 1985; Britton et al. 2013). We found that the genetic differentiation between pygmy and standard‐size crocodiles was smaller than the genetic differentiation among standard‐size crocodiles' populations sampled at different river basins.

### 4.1 Population Structure and Genetic Distance Using mtDNA Haplotypes

Our results based on mtDNA do not support the hypothesis that pygmy freshwater crocodiles form a separate species from standard‐size crocodiles. Previous work on species delimitation and the genetic species concept in mammals suggests that two groups of individuals can be considered the same species if the Cytb distance between the groups is below 2%; groups in which Cytb distance is between 2% and 11% require further study to determine species delimitation; and groups separated by a Cytb distance above 11% can be considered different species (Bradley and Baker 2001). Although direct comparisons between crocodylians and mammals are inappropriate, no practical guidelines have yet been developed for applying the genetic species concept to reptiles. However, given that crocodylian mitochondrial genomes exhibit evolutionary rates comparable to those of mammals (Janke and Arnason 1997; Eo and DeWoody 2010), it is reasonable to expect similar levels of genetic divergence among species with comparable divergence times.

We observed that the maximum pairwise distance (1.28%) of Cytb haplotypes between pygmy and standard‐size freshwater crocodiles was below the reported minimum uncorrected distance of 5.2% between the species of Central America/Colombian *C. acutus* and *C. rhombifer* and closer to the intra‐species maximum of 0.8% for *C. acutus* within Central America and Colombia (Bloor et al. 2015). Few studies have used Cytb to estimate pairwise distance in crocodylian species, and while more data are available for CR, it is less reliable for identifying speciation. The maximum CR pairwise distance of 1.21% for all pygmy and standard‐size freshwater crocodile haplotypes was within the intra‐species ranges reported in other *Crocodylus* studies, ranging from 0.48% to 1.8% (Gratten 2003; Ray et al. 2004; Luck et al. 2012). Studies that have reported on the interspecific CR pairwise distance of *Crocodylus* species had minimum values of 5.53% (Gratten 2003; Ray et al. 2004). Additionally, the predominant pygmy Cytb and CR haplotypes were both observed in standard‐size populations.

The most common haplotype in standard‐size freshwater crocodiles, Cytb_H1, was not present in pygmy freshwater crocodiles. In contrast, the only haplotype unique to pygmy freshwater crocodiles, Cytb_16, differed from Cytb_H1 by a single mutation. The predominant pygmy freshwater crocodile haplotypes Cytb_H2 and CR_H2 differed from the most common haplotypes by eight and four mutations, respectively, indicating they may be different ancestral lineages. It is important to note that Cytb_H2 and CR_H2 are shared between pygmy and standard‐size freshwater crocodiles, suggesting any diversification between pygmy and standard‐sized freshwater crocodiles may be limited to changes in allele frequencies due to genetic drift. However, these differences may be an artefact of the small sample size. A third lineage may be present in standard‐size populations from the eastern Kimberley area, with a cluster of Cytb haplotypes unique to the area that differ from the common haplotype by eight mutations and a CR haplotype unique to the area that differs from the common haplotype by six mutations. This is a significant finding in determining whether pygmy crocodiles should be considered a separate species, as this standard‐sized population from eastern Kimberley exhibits greater genetic differences. The MJN created from concatenated Cytb and CR sequences supported these conclusions but does not provide any further resolution (Figure A2). Larger sample sizes from all populations could help resolve the dynamics among these populations.

### 4.2 Population Clustering Using dd‐RADseq

The PCA and STRUCTURE graphs supported genetic clustering by river basin rather than by pygmy or standard‐size phenotype. This pattern is consistent with the AMOVA, which detected a relatively high variance component among populations and moderate global structure. Genetic differentiation as measured by *F*_*ST*_ further confirms differentiation is very low within the Ord River Basin (i.e., Argyle/Kununurra), and low between adjacent west Kimberley sites (i.e., Windjana/Geikie), but substantially higher for all comparisons involving Liverpool.

Further genomic studies could also incorporate data from Bullo River pygmy populations to assess whether pygmy freshwater crocodiles share more genetic similarity with the Liverpool River Basin pygmy freshwater crocodiles or with standard‐size freshwater crocodiles in their own Victoria River Basin. There was no evidence of structure within river basins, which may be due to the limited sampling or may represent the true gene flow within river basins. The relatedness matrix and fixed differences indicated restricted gene flow among river basins. This is expected given the limited overland movements and site fidelity behaviors of the freshwater crocodile (Lang 1978; Webb et al. 1983), vast geographical distances (approximately 400–1100 km), and geographic barriers between the sampled basins.

Fixed difference analyses can be used as indicators of a lack of gene flow between populations. While our results do show significant differences in allele fixation among our three geographic areas of west Kimberley area (Lennard and Fitzroy River Basins), west Kimberley area (Ord River Basin), and Arnhem Land escarpments (Liverpool River Basin), our analysis lacked the comprehensive coverage of populations (only one to three sample sites within each river basin) needed to accept this as an accurate representation of OTUs across the freshwater crocodile distribution (Georges et al. 2018). While the east and west Kimberley regions are adjacent to each other, neither is adjacent to the Arnhem Land escarpment area, and even within the Kimberley region, no sample sites were located along the east/west divide where we would most likely see cases of migration and gene flow if they were occurring.

Observed and expected heterozygosities were low in all sample locations. This low heterozygosity, as well as the relatedness matrix and positive inbreeding coefficients across all sites, could be of conservation concern for all Australian freshwater crocodile populations. They may be evidence of a past bottleneck and loss of genetic diversity (Nei et al. 1975) or indicate inbreeding depression and genomic erosion (Bosse and van Loon 2022). The effective population size (N_e_) previously reported for Lake Argyle (Ord River) was lower than expected for a wild population (Cao et al. 2020), however, N_e_ reported at Windjana Gorge (Lennard River) and Geike Gorge (Fitzroy River) was not. Specific conservation concerns and management considerations related to Kimberley region standard‐size crocodiles have already been discussed in Cao et al. (2020), and conservation concerns specific to pygmy populations will be discussed further below.

### 4.3 Evidence for Speciation Potential

While there is little genetic evidence to support a separate taxonomic status between pygmy and standard‐size freshwater crocodiles, the geographic isolation of the latter populations and the phenotypic differences indicate the potential for peripatric speciation. This is a type of allopatric speciation where the secondary population is smaller and, therefore, more likely to experience genetic drift and allele fixation than the first population. We can observe possible evidence of genetic drift in the Cytb and CR haplotype maps, where the predominant haplotype differs among populations, and in the SNP data analyses, where fixed difference analysis and STRUCTURE clusters occur at geographic boundaries. Allopatric speciation is a process rather than the result of a single event in time. It is not always a linear progression from “same species” to “different species” but could instead involve periods of allopatry mixed with periods of gene flow or introgression events (Georges et al. 2018). As a form of allopatric speciation, peripatric speciation encounters all the same challenges for genetic species delimitation and the “burden of proof” required for diagnosing species from OTUs identified using molecular techniques, such as fixed differences, is greater in allopatric scenarios compared to sympatric or parapatric ones (Unmack et al. 2022). For cases where intermediate sample sites exist but have not been included in the analysis—as is the case with our pygmy and standard‐size freshwater crocodiles—the burden of proof is higher still. As such, a greater distribution of sample sites, particularly along the contact zones between geological divisions and putative migration barriers, is needed to provide a more robust analysis of possible speciation.

It is believed that the pygmy freshwater crocodile populations in the Liverpool River and Bullo River escarpment areas exhibit extreme, yet species‐typical, phenotypic plasticity due to resource limitations. Crocodile fitness is partially influenced by the environments they inhabit, with limited food resources known to reduce growth rates and maximum size in reptiles, including crocodiles (Webb and Messel 1978; Stamps and Tanaka 1981; Le Galliard et al. 2005). Mark‐and‐recapture studies across the Kimberley region indicate that, on average, larger body sizes are observed in freshwater crocodiles living in permanent water bodies with a more abundant food supply (Somaweera et al. 2013). For instance, the average body sizes of both male and female crocodiles from Lake Argyle and Lake Kununurra (Ord River system), where water remains deep throughout the year with abundant fish and invertebrate life, are larger than those from the Lennard and Fitzroy River systems, which dries into smaller pools with limited food supply (Figure 1). Even within the Fitzroy River system, crocodiles from lower river sections, where both water and food are available year‐round (e.g., Camballin Weir), tend to be larger than those from the upper reaches (e.g., Mornington) (Somaweera pers. obs.). Although food abundance has not been empirically measured in the Liverpool and Bullo River habitats, field observations suggest a reduction in fish, especially the larger species (Webb 1985), which is consistent with evidence of declining and unstable fish communities in higher‐elevation river systems in the Northern Territory (Bishop et al. 1990).

However, physical barriers (waterfalls, ravines) between the pygmy freshwater crocodiles' escarpment habitats and nearby downstream freshwater crocodile populations also make a case as an isolating mechanism for allopatric speciation. The physical isolation of these pygmy populations, unique haplotypes, private alleles, and genetic structuring indicate that there has been limited gene flow between these populations and the standard‐size freshwater populations. Further studies into the movement of individual animals and more in‐depth molecular analysis may help uncover the extent of this phenomenon.

### 4.4 Implications for Conservation

Small population size and genetic drift can increase vulnerability to environmental and demographic threats. This study demonstrates elevated levels of inbreeding and relatedness across several populations, with particularly high values observed in pygmy freshwater crocodiles. The higher levels of inbreeding in the pygmy populations are of concern as they already face several heightened threats, to which inbreeding and a loss of genetic diversity will likely make them more susceptible. Invasive species such as cane toads ( *Rhinella marina* ) have already been shown to affect the size and demographics of the pygmy freshwater crocodile population at Bullo River (Britton et al. 2013), and it is of pressing concern that the population at Liverpool River be similarly investigated as effects of cane toads can be highly site specific (Somaweera and Shine 2011; Somaweera et al. 2013). The small body size of these crocodiles may also mean that they are more susceptible to seasonal changes in food availability (Britton et al. 2013). This could become more problematic as climate change in the Northern Territory is predicted to increase rainfall, but also to increase periods of drought, temperature, and carbon dioxide levels (Dunlop and Brown 2008), all of which could affect resource availability.

In light of both immediate and long‐term conservation concerns, it is recommended that the pygmy freshwater crocodile be considered an “ecological species”—a designation in which barriers to gene flow arise from ecological factors (Butlin et al. 2012). This classification reflects the species' apparent adaptation to the distinctive climate and resource conditions of its isolated river escarpment habitats. Such unique and often isolated populations of crocodylians are of considerable ecological and historical interest, given their distinctive adaptations for utilising resources in different environments. These adaptations have been observed in several crocodylian species, including Australian freshwater crocodiles (Somaweera et al. 2014; Shirley et al. 2017).

A targeted conservation program could be designed for these unique populations, similar to those implemented for other freshwater crocodile and lizard populations (Somaweera et al. 2013, 2019; De Queiroz and Weins 2007; Ward‐Fear et al. 2024). Conserving these pygmy freshwater crocodile populations is crucial, as they represent the genetic variability that enables freshwater crocodiles to adapt to environmental changes. This adaptability will become increasingly vital to the survival of the species in the face of growing threats of invasive species and climate change (Dunlop and Brown 2008; Britton et al. 2013).

## 5 Conclusions

Maternal and bi‐parental genetic analyses indicate that the pygmy freshwater crocodile population in the upstream Liverpool River escarpment is genetically distinct from standard‐size freshwater crocodile populations. However, the level of differentiation is comparable to that observed among other standard‐size crocodile populations, some of which may also be experiencing genetic isolation. As such, the pygmy freshwater crocodiles should be regarded as part of the same species as other standard‐sized populations. Nonetheless, further research is needed to understand better the dynamics of this population, including studies on the pygmy crocodiles at Bullo River, a broader sampling distribution, more extensive genetic markers, and a closer examination of individual dispersal patterns. This would provide valuable insights into isolation, gene flow, and the processes of speciation. In the meantime, the pygmy freshwater crocodiles should be viewed as a unique “ecological species,” whose isolation and environmental adaptability make them particularly important to conserve in the face of potential threats.

## Author Contributions

**Katherine Brittain:** conceptualization (equal), data curation (equal), formal analysis (equal), methodology (supporting), project administration (equal), writing – original draft (lead), writing – review and editing (equal). **Torre Muhlbach:** data curation (equal). **Rui Cao:** data curation (equal). **Jose Luis Mijangos:** formal analysis (equal), methodology (supporting), writing – review and editing (supporting). **Dean Yibarbuk:** resources (supporting). **Erin O'Brien:** resources (equal). **Ruchira Somaweera:** resources (equal), writing – review and editing (equal). **Nancy N. FitzSimmons:** data curation (equal), resources (equal), writing – review and editing (equal). **Jaime Gongora:** conceptualization (equal), methodology (lead), project administration (equal), supervision (lead), writing – review and editing (equal).

## Conflicts of Interest

The authors declare no conflicts of interest.

## Supporting information


**Table A1.** Locations and numbers of *Crocodylus johnstoni* samples used for each analysis.
**Figure A2**. Median joining network of concatenated cytochrome b and control region haplotypes from 85 *Crocodylus johnstoni* samples across 13 river basins. Pygmy sized samples from Liverpool River Basin are represented in purple. Mutations between haplotypes are represented by a dash.

## Data Availability

All Cytochrome B sequences can be found in the BankIt database with GenBank accession numbers PV102152 to PV102236. All Control Region sequences can be found in the BankIt database with accession numbers PV102049 to PV102151. Raw reads from standard‐size Australian freshwater crocodiles are stored in the Sequence Read Archive under project number PRJNA551392.

## References

[ece374055-bib-0001] Afsharian, A. , M. R. Ā. Naṣīrī , E. Ebrahimi , and A. Javadmanesh . 2018. “Genetic Diversity of Persian Crocodile *Crocodylus palustris* Using Sequencing of D‐Loop and Cyt b Regions of Mitochondria.” Biyutiknuluzhī‐i Kishāvarzī 9: 17–38.

[ece374055-bib-0002] Baker, R. J. , and R. D. Bradley . 2006. “Speciation in Mammals and the Genetic Species Concept.” Journal of Mammalogy 87: 643–662. 19890476 10.1644/06-MAMM-F-038R2.1 PMC2771874

[ece374055-bib-0003] Bishop, K. A. , S. A. Allen , D. A. Pollard , and M. G. Cook . 1990. “Ecological Studies in the Freshwater Fishes of the Alligator Rivers Region, Northern Territory.”

[ece374055-bib-0004] Bloor, P. , C. Ibáñez , and T. A. Viloria‐Lagares . 2015. “Mitochondrial DNA Analysis Reveals Hidden Genetic Diversity in Captive Populations of the Threatened American Crocodile ( *Crocodylus acutus* ) in Colombia.” Ecology and Evolution 5: 130–140. 25628870 10.1002/ece3.1307 PMC4298440

[ece374055-bib-0005] Bosse, M. , and S. van Loon . 2022. “Challenges in Quantifying Genome Erosion for Conservation.” Frontiers in Genetics 13: 960958. 36226192 10.3389/fgene.2022.960958 PMC9549127

[ece374055-bib-0006] Bradley, R. D. , and R. J. Baker . 2001. “A Test of the Genetic Species Concept: Cytochrome‐b Sequences and Mammals.” Journal of Mammalogy 82: 960–973. 10.1644/06-MAMM-F-038R2.1 PMC2771874 19890476

[ece374055-bib-0007] Britton, A. R. C. , E. K. Britton , and C. R. McMahon . 2013. “Impact of a Toxic Invasive Species on Freshwater Crocodile (*Crocodylus johnstoni*) Populations in Upstream Escarpments.” Wildlife Research 40: 312–317.

[ece374055-bib-0008] Butlin, R. , A. Debelle , C. Kerth , et al. 2012. “What Do We Need to Know About Speciation?” Trends in Ecology & Evolution (Amsterdam) 27: 32–44. 10.1016/j.tree.2011.09.002 21978464

[ece374055-bib-0009] Cao, R. , R. Somaweera , K. Brittain , N. Fitzsimmons , A. Georges , and J. Gongora . 2020. “Genetic Structure and Diversity of Australian Freshwater Crocodiles ( *Crocodylus johnstoni* ) From the Kimberley, Western Australia.” Conservation Genetics 21: 421–429.

[ece374055-bib-0010] Catchen, J. , P. A. Hohenlohe , S. Bassham , A. Amores , and W. A. Cresko . 2013. “Stacks: An Analysis Tool Set for Population Genomics.” Molecular Ecology 22: 3124–3140. 23701397 10.1111/mec.12354 PMC3936987

[ece374055-bib-0012] De Queiroz, K. , and J. Weins . 2007. “Species Concepts and Species Delimitation.” Systematic Biology 56: 879–886. 18027281 10.1080/10635150701701083

[ece374055-bib-0013] Dray, S. , and A.‐B. Dufour . 2007. “The ade4 Package: Implementing the Duality Diagram for Ecologists.” Journal of Statistical Software 22: 1–20.

[ece374055-bib-0014] Dunlop, M. , and P. R. Brown . 2008. Implications of Climate Change for Australia's National Reserve System: A Preliminary Assessment. Dept. of Climate Change.

[ece374055-bib-0015] Earl, D. A. , and B. M. vonHoldt . 2012. “STRUCTURE HARVESTER: A Website and Program for Visualizing STRUCTURE Output and Implementing the Evanno Method.” Conservation Genetics Resources 4: 359–361.

[ece374055-bib-0016] Edwards, G. P. , G. J. Webb , S. C. Manolis , and A. Mazanov . 2017. “Morphometric Analysis of the Australian Freshwater Crocodile ( *Crocodylus johnstoni* ).” Australian Journal of Zoology 65: 97–111.

[ece374055-bib-0017] Endelman, J. B. 2011. “Ridge Regression and Other Kernels for Genomic Selection With R Package rrBLUP.” Plant Genome 4: 250–255.

[ece374055-bib-0018] Endelman, J. B. , and J.‐L. Jannink . 2012. “Shrinkage Estimation of the Realized Relationship Matrix.” G3: Genes, Genomes, Genetics 2: 1405–1413. 23173092 10.1534/g3.112.004259 PMC3484671

[ece374055-bib-0019] Eo, S. H. , and J. A. DeWoody . 2010. “Evolutionary Rates of Mitochondrial Genomes Correspond to Diversification Rates and to Contemporary Species Richness in Birds and Reptiles.” Proceedings of the Royal Society B: Biological Sciences 277: 3587–3592. 10.1098/rspb.2010.0965 PMC2982251 20610427

[ece374055-bib-0020] Evanno, G. , S. Regnaut , and J. Goudet . 2005. “Detecting the Number of Clusters of Individuals Using the Software Structure: A Simulation Study.” Molecular Ecology 14: 2611–2620. 15969739 10.1111/j.1365-294X.2005.02553.x

[ece374055-bib-0021] Excoffier, L. , P. E. Smouse , and J. M. Quattro . 1992. “Analysis of Molecular Variance Inferred From Metric Distances Among DNA Haplotypes: Application to Human Mitochondrial DNA Restriction Data.” Genetics (Austin) 131: 479–491. 1644282 10.1093/genetics/131.2.479 PMC1205020

[ece374055-bib-0022] FitzSimmons, N. , J. C. Buchan , P. V. Lam , et al. 2002. “Identification of Purebred *Crocodylus siamensis* for Reintroduction in Vietnam.” Journal of Experimental Zoology 294: 373–381. 12461816 10.1002/jez.10201

[ece374055-bib-0023] FitzSimmons, N. , S. Tanksley , M. R. J. Forstner , et al. 2000. “Microsatellite Markers for *Crocodylus*: New Genetic Tools for Population Genetics, Mating System Studies and Forensics.” In Crocodilian Biology and Evolution, edited by G. Grigg , F. Seebacher , and C. Franklin . Surrey Beatty & Sons.

[ece374055-bib-0024] Georges, A. , B. Gruber , G. B. Pauly , et al. 2018. “Genomewide SNP Markers Breathe New Life Into Phylogeography and Species Delimitation for the Problematic Short‐Necked Turtles (Chelidae: Emydura) of Eastern Australia.” Molecular Ecology 27: 5195–5213. 30403418 10.1111/mec.14925

[ece374055-bib-0025] Gratten, J. 2003. The Molecular Systematics, Phylogeography and Population Genetics of Indo‐Pacific Crocodylus. University of Queensland.

[ece374055-bib-0026] Green, M. R. , and J. Sambrook . 2012. Molecular Cloning: A Laboratory Manual. 4th ed. Cold Spring Harbor Laboratory Press.

[ece374055-bib-0027] Grigg, G. 2015. Biology and Evolution of Crocodylians. Comstock Publishing Associates.

[ece374055-bib-0028] Gruber, B. , P. J. Unmack , O. F. Berry , and A. Georges . 2018. “Dartr: An r Package to Facilitate Analysis of SNP Data Generated From Reduced Representation Genome Sequencing.” Molecular Ecology Resources 18: 691–699. 29266847 10.1111/1755-0998.12745

[ece374055-bib-0029] Hall, T. 1999. “BioEdit: A User‐Friendly Biological Sequence Editor and Analysis Program for Windows 95/98/NT.” Nucleic Acids Symposium Series 41: 95–98.

[ece374055-bib-0030] Hillis, D. M. 2019. “Species Delimitation in Herpetology.” Journal of Herpetology 53: 3–12.

[ece374055-bib-0031] Huang, Y. , C.‐Q. Zhang , and D.‐Z. Li . 2009. “Low Genetic Diversity and High Genetic Differentiation in the Critically Endangered Omphalogramma Souliei (Primulaceae): Implications for Its Conservation.” Journal of Systematics and Evolution 47: 103–109.

[ece374055-bib-0032] Isberg, S. , S. A. Balaguera‐Reina , and J. P. Ross . 2017. *Crocodylus johnstoni* . IUCN Red List of Threatened Species.

[ece374055-bib-0033] Jakobsson, M. , and N. A. Rosenberg . 2007. “CLUMPP: A Cluster Matching and Permutation Program for Dealing With Label Switching and Multimodality in Analysis of Population Structure.” Bioinformatics 23: 1801–1806. 17485429 10.1093/bioinformatics/btm233

[ece374055-bib-0034] Janke, A. , and U. Arnason . 1997. “The Complete Mitochondrial Genome of Alligator Mississippiensis and the Separation Between Recent Archosauria (Birds and Crocodiles).” Molecular Biology and Evolution 14: 1266–1272. 9402737 10.1093/oxfordjournals.molbev.a025736

[ece374055-bib-0035] Kamvar, Z. N. , J. F. Tabima , and N. J. Grünwald . 2014. “Poppr: An R Package for Genetic Analysis of Populations With Clonal, Partially Clonal, and/or Sexual Reproduction.” PeerJ (San Francisco, CA) 2: e281. 10.7717/peerj.281 PMC3961149 24688859

[ece374055-bib-0036] Keller, M. C. , P. M. Visscher , and M. E. Goddard . 2011. “Quantification of Inbreeding due to Distant Ancestors and Its Detection Using Dense Single Nucleotide Polymorphism Data.” Genetics (Austin) 189: 237–249. 21705750 10.1534/genetics.111.130922 PMC3176119

[ece374055-bib-0037] Kumar, S. , G. Stecher , M. Li , C. Knyaz , and K. Tamura . 2018. “MEGA X: Molecular Evolutionary Genetics Analysis Across Computing Platforms.” Molecular Biology and Evolution 35: 1547–1549. 29722887 10.1093/molbev/msy096 PMC5967553

[ece374055-bib-0038] Labarre, D. , P. Charruau , S. G. Platt , T. R. Rainwater , J. R. Cedeño‐Vázquez , and H. González‐Cortés . 2017. “Morphological Diversity of the American Crocodile ( *Crocodylus acutus* ) in the Yucatán Peninsula.” Zoomorphology 136: 387–401.

[ece374055-bib-0039] Lang, J. W. 1978. “Crocodilian Behaviour: Implications for Management.” In Wildlife Management: Crocodiles and Alligators, edited by G. Webb , S. C. Manolis , and P. Whitehead . Surrey Beatty & Sons in association with the Conservation Commission of the Northern Territory.

[ece374055-bib-0040] Le Galliard, J. F. , R. Ferrière , and J. Clobert . 2005. “Juvenile Growth and Survival Under Dietary Restriction: Are Males and Females Equal?” Oikos 111: 368–376.

[ece374055-bib-0041] Leigh, J. W. , and D. Bryant . 2015. “Popart: Full‐Feature Software for Haplotype Network Construction.” Methods in Ecology and Evolution 6: 1110–1116.

[ece374055-bib-0042] Luck, N. L. , K. C. Thomas , V. E. Morin‐Adeline , et al. 2012. “Mitochondrial DNA Analyses of the Saltwater Crocodile (*Crocodylus porosus*) From the Northern Territory of Australia.” Australian Journal of Zoology 60: 18–25.

[ece374055-bib-0043] Mayden, R. L. 1999. “Consilience and a Hierarchy of Species Concepts: Advances Toward Closure on the Species Puzzle.” Journal of Nematology 31: 95–116. 19270881 PMC2620363

[ece374055-bib-0044] McCue, F. 2014. The Northern Territory's Pygmy Freshwater Crocs Spark Big Debate. NT News.

[ece374055-bib-0045] Meganathan, P. , B. Dubey , and I. Haque . 2009. “Molecular Identification of Crocodile Species Using Novel Primers for Forensic Analysis.” Conservation Genetics 10: 767–770.

[ece374055-bib-0046] Meredith, R. W. , E. R. Hekkala , G. Amato , and J. Gatesy . 2011. “A Phylogenetic Hypothesis for Crocodylus (Crocodylia) Based on Mitochondrial DNA: Evidence for a Trans‐Atlantic Voyage From Africa to the New World.” Molecular Phylogenetics and Evolution 60: 183–191. 21459152 10.1016/j.ympev.2011.03.026

[ece374055-bib-0047] Mijangos, J. L. , B. Gruber , O. Berry , C. Pacioni , and A. Georges . 2022. “dartR v2: An Accessible Genetic Analysis Platform for Conservation, Ecology and Agriculture.” Methods in Ecology and Evolution 13: 2150–2158.

[ece374055-bib-0048] Naomi, S.‐I. 2011. “On the Integrated Frameworks of Species Concepts: Mayden's Hierarchy of Species Concepts and de Queiroz's Unified Concept of Species.” Journal of Zoological Systematics and Evolutionary Research 49: 177–184.

[ece374055-bib-0049] Nei, M. 1987. Molecular Evolutionary Genetics. Columbia University Press.

[ece374055-bib-0050] Nei, M. , T. Maruyama , and R. Chakraborty . 1975. “The Bottleneck Effect and Genetic Variability in Populations.” Evolution 29: 1–10. 28563291 10.1111/j.1558-5646.1975.tb00807.x

[ece374055-bib-0051] Nestler, J. H. 2012. A Geometric Morphometric Analysis of *Crocodylus niloticus* : Evidence for a Cryptic Species Complex. University of Iowa.

[ece374055-bib-0052] Oaks, J. R. 2011. “A Time‐Calibrated Species Tree of Crocodylia Reveals a Recent Radiation of the True Crocodiles.” Evolution 65: 3285–3297. 22023592 10.1111/j.1558-5646.2011.01373.x

[ece374055-bib-0053] Pearman, W. S. , L. Urban , and A. Alexander . 2022. “Commonly Used Hardy–Weinberg Equilibrium Filtering Schemes Impact Population Structure Inferences Using RADseq Data.” Molecular Ecology Resources 22: 2599–2613. 35593534 10.1111/1755-0998.13646 PMC9541430

[ece374055-bib-0054] Pfennig, D. W. , and M. J. West‐Eberhard . 2021. Phenotypic Plasticity & Evolution: Causes, Consequences, Controversies. 1st ed. CRC Press.

[ece374055-bib-0055] Pritchard, J. K. , M. Stephens , and P. Donnelly . 2000. “Inference of Population Structure Using Multilocus Genotype Data.” Genetics 155: 945–959. 10835412 10.1093/genetics/155.2.945 PMC1461096

[ece374055-bib-0056] Pritchard, J. K. , and W. Wen . 2002. Documentation for Structure Software: Version 2. University of Chicago Press.

[ece374055-bib-0057] Purcell, S. , B. Neale , K. Todd‐Brown , et al. 2007. “PLINK: A Tool Set for Whole‐Genome Association and Population‐Based Linkage Analyses.” American Journal of Human Genetics 81: 559–575. 17701901 10.1086/519795 PMC1950838

[ece374055-bib-0058] Ray, D. , J. Dever , S. Platt , et al. 2004. “Low Levels of Nucleotide Diversity in Crocodylus Moreletii and Evidence of Hybridization With *C. acutus* .” Conservation Genetics 5: 449–462.

[ece374055-bib-0059] Read, M. , B. Wright , and C. Enoch . 2004. “Crocodiles of Queensland.” In 17th Working Meeting of the Crocodile Specialist Group. IUCN ‐ The World Conservation Union.

[ece374055-bib-0060] Rosenberg, N. A. 2004. “Distruct: A Program for the Graphical Display of Population Structure.” Molecular Ecology Notes 4: 137–138.

[ece374055-bib-0061] Shirley, M. H. , B. Burtner , R. Oslisly , D. Sebag , and O. Testa . 2017. “Diet and Body Condition of Cave‐Dwelling Dwarf Crocodiles ( *Osteolaemus tetraspis* , Cope 1861) in Gabon.” African Journal of Ecology 55: 411–422.

[ece374055-bib-0062] Somaweera, R. , M. L. Brien , T. Sonneman , R. K. Didham , and B. L. Webber . 2019. “Absence of Evidence Is Not Evidence of Absence: Knowledge Shortfalls Threaten the Effective Conservation of Freshwater Crocodiles.” Global Ecology and Conservation 20: e00773.

[ece374055-bib-0063] Somaweera, R. , and R. Shine . 2011. “The (Non) Impact of Invasive Cane Toads on Freshwater Crocodiles at Lake Argyle in Tropical Australia.” Animal Conservation 15: 152–163.

[ece374055-bib-0064] Somaweera, R. , R. Shine , J. Webb , T. Dempster , and M. Letnic . 2013. “Why Does Vulnerability to Toxic Invasive Cane Toads Vary Among Populations of Australian Freshwater Crocodiles?” Animal Conservation 16: 86–96.

[ece374055-bib-0065] Somaweera, R. , D. Woods , and T. Sonneman . 2014. “A Note on the Australian Freshwater Crocodiles Inhabiting Tunnel Creek Cave, West Kimberley.” Records of the Western Australian Museum 29: 82.

[ece374055-bib-0066] Stamps, J. , and S. Tanaka . 1981. “Influence of Food and Water on Growth Rates in a Tropical Lizard ( *Anolis aeneus* ).” Ecology 62: 33–40.

[ece374055-bib-0068] Thompson, J. , D. Higgins , and T. Gibson . 1994. “CLUSTAL W: Improving the Sensitivity of Progressive Multiple Sequence Alignment Through Sequence Weighting, Position‐Specific Gap Penalties and Weight Matrix Choice.” Nucleic Acids Research 22: 4673–4680. 7984417 10.1093/nar/22.22.4673 PMC308517

[ece374055-bib-0069] Tucker, A. , C. Limpus , K. McDonald , and H. McCallum . 2006. “Growth Dynamics of the Freshwater Crocodile ( *Crocodylus johnstoni* ) in the Lynd River, Queensland.” Australian Journal of Zoology 54: 409–415.

[ece374055-bib-0070] Uetz, P. , P. Freed , P. Aguilar , F. Reyes , J. Kudera , and J. Hošek . 2025. “The Reptile Database.”

[ece374055-bib-0071] Unmack, P. J. , M. Adams , M. P. Hammer , et al. 2022. “Plotting for Change: An Analytical Framework to Aid Decisions on Which Lineages Are Candidate Species in Phylogenomic Species Discovery.” Biological Journal of the Linnean Society 135: 117–137.

[ece374055-bib-0072] Ward‐Fear, G. , M. Bruny , t. B. Rangers , C. Forward , I. Cooksey , and R. Shine . 2024. “Taste Aversion Training Can Educate Free‐Ranging Crocodiles Against Toxic Invaders.” Proceedings of the Royal Society B: Biological Sciences 291: 20232507. 10.1098/rspb.2023.2507 PMC11321852 39137886

[ece374055-bib-0073] Webb, G. 1985. “Survey of a Pristine Population of Freshwater Crocodiles in the Liverpool River, Arnhem Land, Australia.” National Geographic Society Reports 1979: 841–852.

[ece374055-bib-0074] Webb, G. , and S. C. Manolis . 1989. Crocodiles of Australia. New Holland.

[ece374055-bib-0075] Webb, G. J. W. , S. C. Manolis , and R. Buckworth . 1983. “ *Crocodylus johnstoni* In the McKinlay River Area N. T, III. Growth, Movement and the Population Age Structure.” Wildlife Research 10: 383–401.

[ece374055-bib-0076] Webb, G. J. W. , and H. Messel . 1978. “Morphometric Analysis of *Crocodylus porosus* From the North Coast of Arnhem Land, Northern Australia.” Australian Journal of Zoology 26: 1–27.

[ece374055-bib-0077] Webber, K. 2015. Zoologist Turns to Crowdfunding for Pygmy Croc Research. ABC News.

[ece374055-bib-0078] West‐Eberhard, M. J. 2005. “Developmental Plasticity and the Origin of Species Differences.” Proceedings of the National Academy of Sciences of the United States of America 102: S6543. 10.1073/pnas.0501844102 PMC1131862 15851679

[ece374055-bib-0079] Yang, J. , S. H. Lee , M. E. Goddard , and P. M. Visscher . 2011. “GCTA: A Tool for Genome‐Wide Complex Trait Analysis.” American Journal of Human Genetics 88: 76–82. 21167468 10.1016/j.ajhg.2010.11.011 PMC3014363

[ece374055-bib-0080] Zachos, F. E. 2016. Species Concepts in Biology: Historical Development, Theoretical Foundations and Practical Relevance. 1st ed. Springer International Publishing.

