# Supplementary material for: Unique Population or Unique Species? Genetic Insights Into the Pygmy Freshwater Crocodiles of Northern Australia
Source: Ecol Evol. 2026 Jul 31;16(8):e74055. doi: 10.1002/ece3.74055 (PMC13426028; doi:10.1002/ece3.74055)
Supplement: Supplementary file 1 — Table A1. Locations and numbers of Crocodylus johnstoni samples used for each analysis. Figure A2. Median joining network of concatenated cytochrome b and control region haplotypes from 85 Crocodylus johnstoni samples across 13 river basins. Pygmy sized samples from Liverpool River Basin are represented in purple. Mutations between haplotypes are represented by a dash. [file ECE3-16-e74055-s001.docx]

Appendix 1

*Table A1*: Locations and numbers of Crocodylus johnstoni samples used for each analysis.

| **Sample location** | **No. of samples used in mtDNA analysis** | **No. of samples used in dd-RADseq** | **River Basin** | **Region  (used in dd-RADseq analysis)** | **State** |
| --- | --- | --- | --- | --- | --- |
| King River | 2 | - | Ord | N/A | WA |
| Lake Kununurra | 1 | 6 | Ord | East Kimberley | WA |
| Lake Argyle | 6 | 34 | Ord | East Kimberley | WA |
| Windjana Gorge | 8 | 97 | Lennard | West Kimberley | WA |
| Geike Gorge | 3 | 34 | Fitzroy | West Kimberley | WA |
| Drysdale River | 5 | - | Drysdale | N/A | WA |
| Liverpool River-pygmy | 11 | 8 | Liverpool | Arnhem Land escarpments | NT |
| Mann River | 2 | - | Liverpool | N/A | NT |
| Mary River | 7 | - | Mary | N/A | NT |
| Victoria River | 2 | - | Victoria | N/A | NT |
| Bullo River-pygmy | 18 (CR only) | - | Victoria | N/A | NT |
| Roper River | 9 | - | Roper | N/A | NT |
| Hodgson | 5 | - | Roper | N/A | NT |
| Daly River | 5 | - | Daly | N/A | NT |
| Douglas River | 1 | - | Daly | N/A | NT |
| McKinlay River | 10 | - | Mary | N/A | NT |
| Louis Creek | 1 | - | Gilbert | N/A | QLD |
| Townsville | 1 | - | Ross | N/A | QLD |
| Lakefield National Park | 5 | - | Normanby | N/A | QLD |
| Lawn Hill | 1 | - | Nicholson | N/A | QLD |

Appendix 2


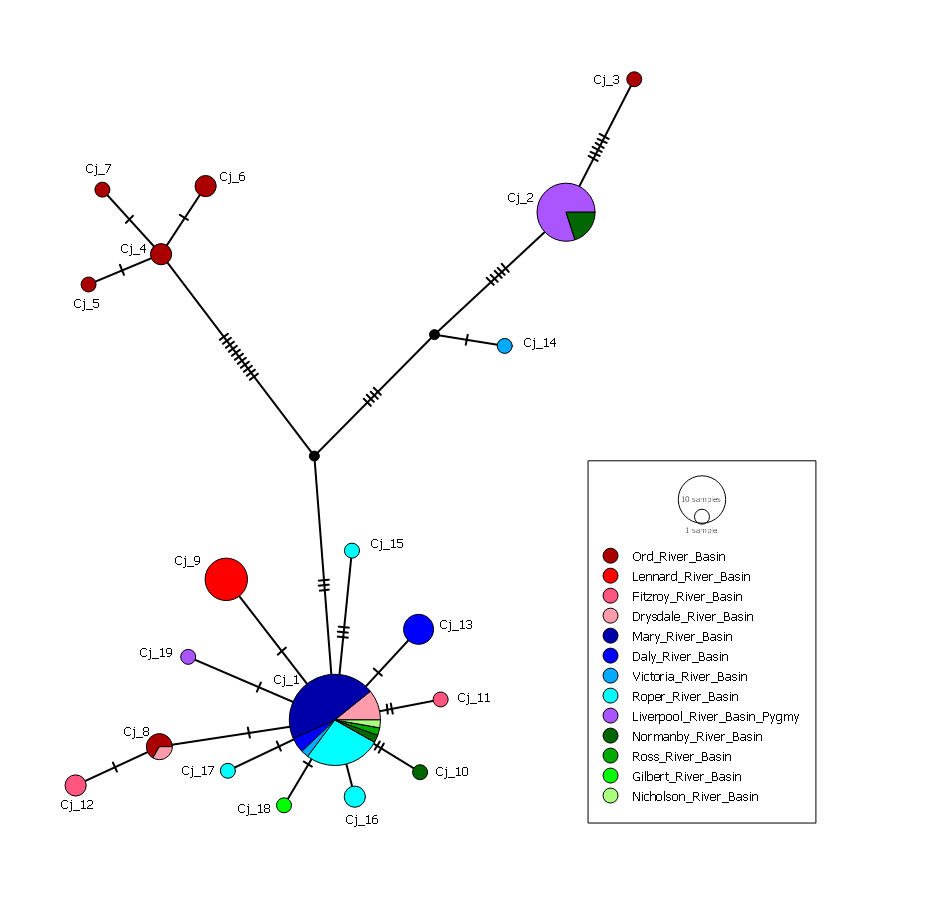


*Figure A2*: Median joining network of concatenated cytochrome b and control region haplotypes from 85 Crocodylus johnstoni samples across 13 river basins. Pygmy sized samples from Liverpool River Basin are represented in purple. Mutations between haplotypes are represented by a dash.
